# Novel application of simultaneous multi-image display during complex robotic abdominal procedures

**DOI:** 10.1186/1471-2482-14-13

**Published:** 2014-03-15

**Authors:** Yanghee Woo, Gi Hong Choi, Byung Soh Min, Woo Jin Hyung

**Affiliations:** 1 Department of Surgery, College of Physicians and Surgeons, Columbia University, New York NY, USA; 2 Department of Surgery, Yonsei University College of Medicine, 134 Shinchon-dong Seodaemun-gu, Seoul 120-752, Republic of Korea; 3 Robot and MIS Center, Yonsei University College of Medicine, Seoul, Korea

**Keywords:** Multi-image display, Intraoperative endoscopy, Robotic surgery, Minimally invasive surgery

## Abstract

**Background:**

The surgical robot offers the potential to integrate multiple views into the surgical console screen, and for the assistant’s monitors to provide real-time views of both fields of operation. This function has the potential to increase patient safety and surgical efficiency during an operation. Herein, we present a novel application of the multi-image display system for simultaneous visualization of endoscopic views during various complex robotic gastrointestinal operations.

All operations were performed using the da Vinci Surgical System (Intuitive Surgical, Sunnyvale, CA, USA) with the assistance of Tilepro, multi-input display software, during employment of the intraoperative scopes. Three robotic operations, left hepatectomy with intraoperative common bile duct exploration, low anterior resection, and radical distal subtotal gastrectomy with intracorporeal gastrojejunostomy, were performed by three different surgeons at a tertiary academic medical center.

**Results:**

The three complex robotic abdominal operations were successfully completed without difficulty or intraoperative complications. The use of the Tilepro to simultaneously visualize the images from the colonoscope, gastroscope, and choledochoscope made it possible to perform additional intraoperative endoscopic procedures without extra monitors or interference with the operations.

**Conclusion:**

We present a novel use of the multi-input display program on the da Vinci Surgical System to facilitate the performance of intraoperative endoscopies during complex robotic operations. Our study offers another potentially beneficial application of the robotic surgery platform toward integration and simplification of combining additional procedures with complex minimally invasive operations.

## Background

The advantages of a robotic surgery platform have significant impact on the ability of surgeons to perform complex procedures. Currently, surgical robots provide advantages in two general areas: image-guided procedures and surgeon-controlled robotic operations [1]. Neurosurgery and orthopedics have successfully employed image-guided and computer assistance in their operations to increase both surgical accuracy and patient safety [2-4]. The use of surgical robots in general surgery has primarily been limited to surgeon-manipulated robotic operations, which have also demonstrated improved patient outcome [5-11]. As surgeons gain experience in robotic abdominal operations, surgeons are able to maximize the translation of technologies incorporated into the robot for patient benefit.

Complex robotic hepatobiliary, colon, and gastric surgeries have been successfully performed using the da Vinci Surgical System (Intuitive Surgical, Sunnyvale, CA) [12-16]. At times, additional intraoperative procedures to acquire more information on the patient’s disease are required to supplement the preoperative work-up. For example, patients with gastrointestinal tumors may need intraoperative endoscopic localization of the tumors. During a liver resection for intrahepatic cholelithiasis common bile duct exploration, the use of a choledochoscope is required. These operations are being performed, in most cases, using additional monitors that make simultaneous examination of the intraabdominal working space and endoscopy difficult for a single surgeon. The surgical robot offers the potential to integrate multiple views into the surgical console screen, and for the assistant’s monitors to provide real-time views of both fields of operation. This function has the potential to increase patient safety and surgical efficiency during an operation.

Our report presents a novel application of the da Vinci Surgical System’s Tilepro multi-display system for simultaneous visualization of endoscopic views during various complex robotic gastrointestinal operations.

## Methods

**Figure 1:**
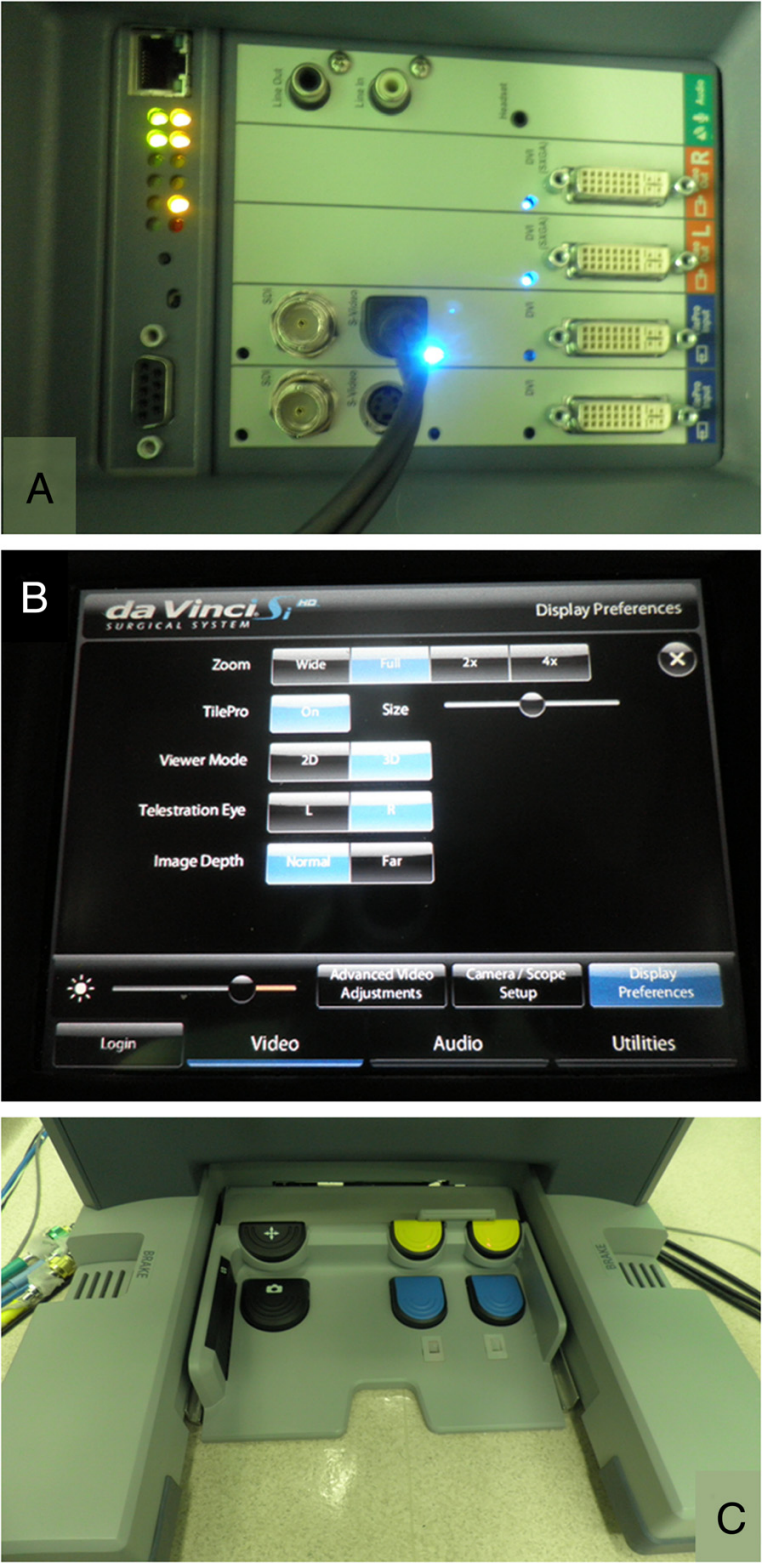
**Tilpro™ set up. (A)** The posterior view of the da Vinci Surgical System input device for endoscopic video output. The surgeon can control the video input from the endoscopic procedures from the surgeon’s console using the touch screen of the control bar **(B)** and the camera foot pedal **(C)**.

All operations were performed using the da Vinci Surgical System (Intuitive Surgical, Sunnyvale, CA, USA) with the assistance of Tilepro, multi-input display software, during employment of the intraoperative scopes. Tilepro software integrates into the robotic platform and permits the surgeon and operating room personnel to view up to three different images; that is, the operative view and images from two different video sources, simultaneously. Before each operation, the video output from the HDTV-compatible CV-180 Video Processor (Olympus, Tokyo, Japan), used for the three endoscopic procedures, was connected to the da Vinci Surgical System’s console (Figure 1A). During intraoperative endoscopy, the surgeon activated the endoscopic images by turning on the multi-input Tilepro program at the surgeon’s console using the video panel (Figure 1B). The surgeon was able to switch the endoscopic images on and off by tapping the camera foot pedal (Figure 1C).

### Operative procedures using Tilepro with intraoperative endoscopy

Three robotic operations, left hepatectomy with intraoperative common bile duct exploration, low anterior resection, and radical distal subtotal gastrectomy with intracorporeal gastrojejunostomy, were performed by three different surgeons at a tertiary academic medical center. The patients’ perioperative factors and clinicopathologic characteristics are shown in Table 1.

**Table 1:** Patient characteristics and perioperative factors

**Operation**	**Low anterior resection & colonoscopy**	**Radical gastrectomy & gastroscopy**	**Left hepatectomy & CBDE**
Age (years)	66	41	57
Gender	Female	Male	Female
Comorbidity	HTN	None	Hypothyroidism
Operation time (min)	396	200	465
Blood loss (cc)	500	11	150
Complications	None	None	Fluid collect at hepatic resection line
Length of stay (days)	7	5	12

## Results

### Colonoscopy during robotic low anterior resection

**Figure 2:**
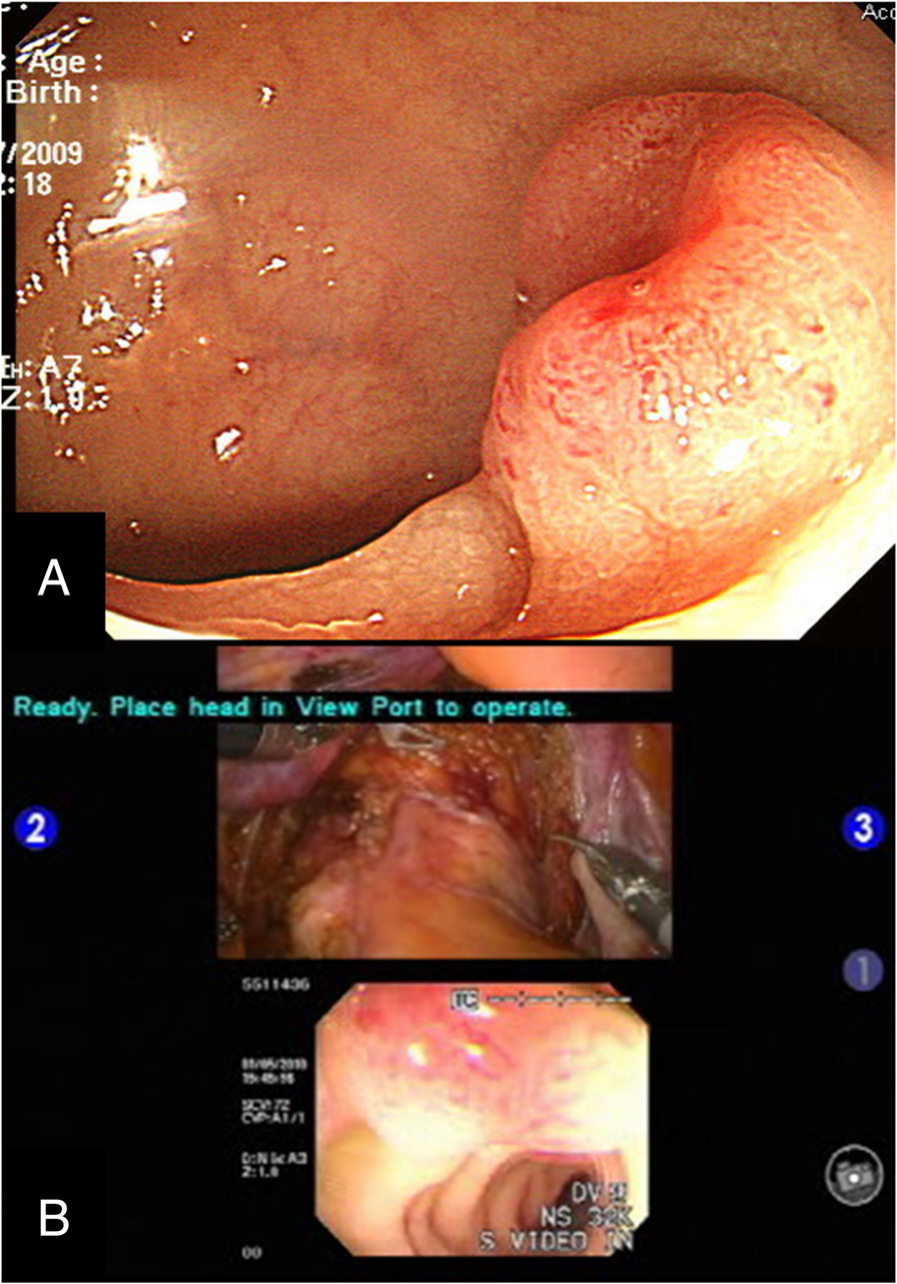
**Preoperative colonoscopy and multi-input view of intraoperative colonoscopy. (A)** The preoperative colonoscopic image. **(B)** Images from the intraoperative colonoscopy for tumor localization are visualized simultaneously with the intraabdominal extraluminal view for accurate determination of the distal rectal margin.

A 66-year-old female patient with hypertension was diagnosed on routine screening colonoscopy with rectal cancer and subsequently underwent robotic low anterior resection with intraoperative colonoscopy. The preoperative colonoscopy revealed an ulcerofungating tumor located in the proximal rectum (Figure 2A). A computed tomography (CT) scan demonstrated no distant metastases.

Robotic low anterior resection was performed using the 6-port method as previously described [17]. In brief, after placement of the ports (one 12-mm and five 8-mm ports) and docking of the robot, a monopolar device, Cadiere forceps, and ultrasonic shears were used to mobilize the splenic flexure. The robotic arms were repositioned for the remainder of the operation. The surgeon mobilized the colon from the lateral peritoneal attachments by division of the left paracolic gutter with careful preservation of the ureteral and gonadal vessels. Under direct 3-dimensional (3D) magnified vision, pelvic autonomic nerves and visceral fascia were preserved. The mesocolon was divided with ligation of the embedded feeding vessels, and the pericolic fat removed for preparation of resection of the sigmoid colon.

At this time, the assistant performed a colonoscopy (Olympus, Optical Co. Ltd, Tokyo, Japan) to localize the rectal lesion. The images from the colonoscopy were displayed on the surgeon’s console along with the intraoperative extraluminal view of the rectum (Figure 2B). The tumor was found on the right side of the mid-rectum, and the distal margin was delineated. Aftern colonoscopic identification of the tumor was confirmed, simultaneous images on the screen of the previously cleared rectum permitted easy identification of the resection margin from the surgeon’s console (Additional file 1). After confirmation of the rectal lesion colonoscope was removed since the patient did not required full colonoscopy.

The operation continued immediately after the colonoscope was removed. The distal margin was resected with an endolinear stapler, and the proximal colon exteriorized. A double stapling method using the CDH 33 mm (Ethicon Endo-surgery, Johnson & Johnson, Cincinnati, OH, USA) was used for the colorectal anastomosis. The total operation time was 396 minutes. The patient was discharged 7 days after surgery without postoperative complications. Final pathological examination revealed a 2.2 × 1.7-cm moderately differentiated adenocarcinoma of the rectum that invaded the proper muscle and one of 24 lymph nodes was positive, staged as pT2N1M0, stage IIIA. The proximal and distal margins from the tumor were 12 cm and 3.3 cm, respectively.

### Gastroscopy during robotic radical gastrectomy

**Figure 3:**
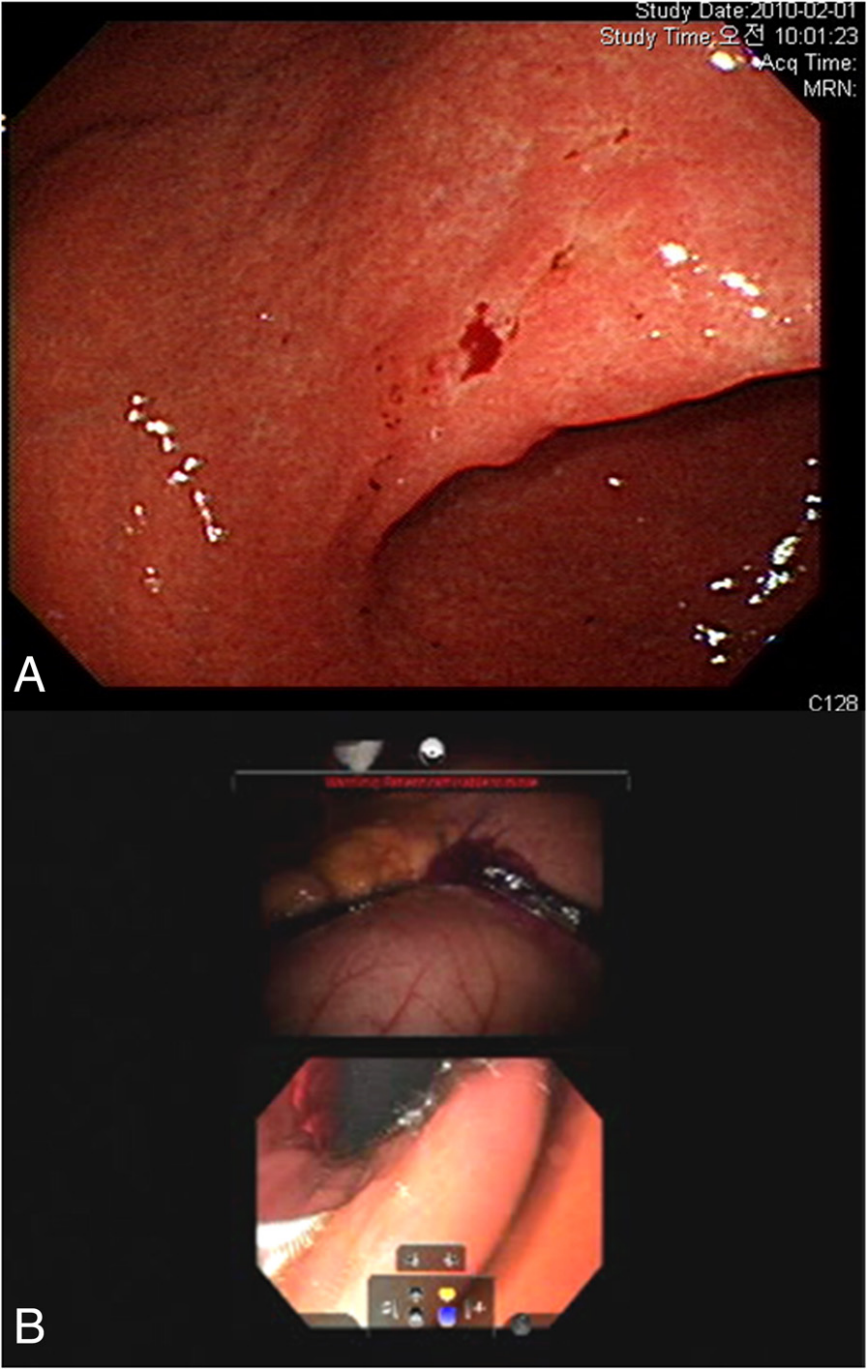
**Preoperative upper endoscopy and multi-input view of intraoperative upper endoscopy.** Gastric cancer was identified on preoperative upper endoscopy **(A)**. During the operation, the surgeon simultaneously viewed the endoscopic and intraabdominal images to accurately determine the proximal gastric resection margin **(B)**.

A 41-year-old male patient with no significant past medical history underwent robotic radical gastrectomy with gastrojejunal reconstruction for early gastric cancer. He was diagnosed with gastric cancer on screening esophagogastroduodenscopy, which was confirmed by biopsy as signet ring carcinoma. The lesion was identified at the angle of the lesser curvature (Figure 3A). A CT scan revealed neither enlarged intraabdominal lymph nodes nor distant metastatic disease.

Robotic radical gastrectomy was performed as previously described [13,18]. In brief, the patient was positioned in the supine position with the operating table placed in reverse Trendelenburg, and the trocars were inserted. After docking of the robotic arms and retraction of the liver, the proximal jejunum was identified and clamped to prevent small bowel distension during endoscopic air insufflation. Endoscopy was performed using a gastroscope (Olympus, Tokyo, Japan) to localize the tumor and determine the proximal resection margin. The endoscopic view was seen simultaneously with the intraoperative view of the stomach (Figure 3B). After endoscopic localization of the tumor, compression of the area of the tumor and the endoscopic light was used to define the proximal margin of resection. This view was facilitated by reducing the light from the robotic camera. The resection line was drawn on the external, anterior wall of the stomach (Additional file 2).

Robotic gastrectomy proceeded with partial omentectomy, ligation and division of the left gastroepiploic vessels, clearance of the greater curvature, and right-side dissection of the head of the pancreas with ligation and division of the right gastroepiploic vessels. After transecting the duodenum, dissection of the soft tissues surrounding the common hepatic artery and the celiac axis continued with identification, ligation, and division of the left gastric artery. The opening of the lesser sac and dissection along the retroperitoneal attachments to the stomach were completed with clearance of the lesser curvature. The previously determined proximal resection line was used as a guide to divide the stomach using an endolinear stapler. Since the intraluminal gastric lesion could not be palpated during robotic surgery, the use of an endoscope to identify the cancer assured an oncologically safe margin of proximal resection for proper intracorporeal anastomosis. Intracorporeal gastrojejunal reconstruction was performed with robotic assistance and stapler manipulation by the bedside assistant.

The total operation time was 200 minutes. The patient had an uneventful postoperative recovery and was discharged 5 days after surgery. Final pathological examination revealed a 3.2-cm × 2.5-cm signet ring cell carcinoma invading the mucosa with no positive lymph nodes, staged as pT1aN0M0, stage IA. Proximal and distal margins from the lesion were 5.5 cm and 4.5 cm, respectively.

### Robot-assisted common bile duct exploration during robotic left hepatectomy

**Figure 4:**
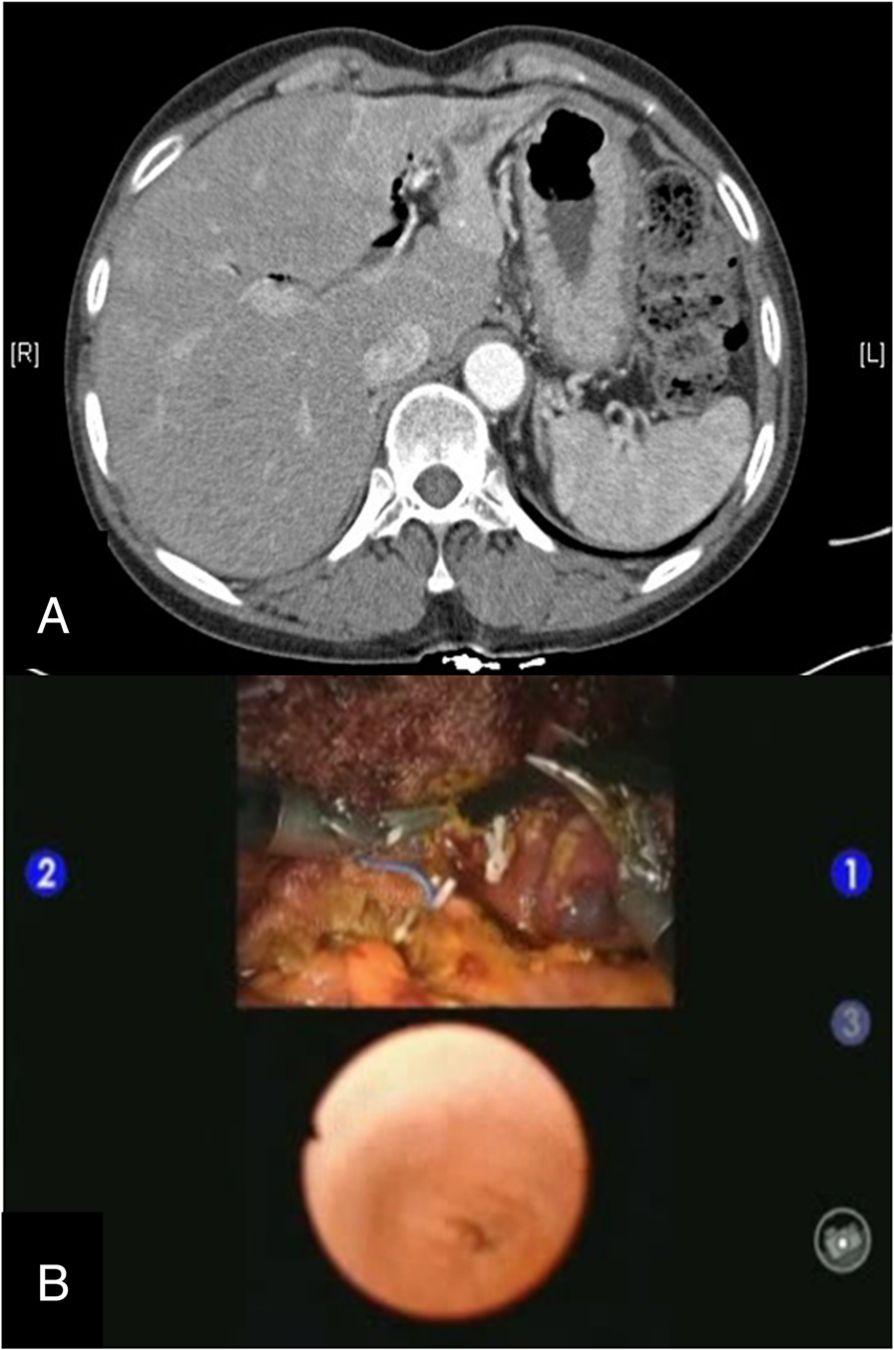
**Images from a preoperative abdominal CT scan and multi-input view of intraoperative common bile duct exploration.** A preoperative CT scan of the patient with cholangiohepatitis shows intrahepatic ductal dilatation and left lobe atrophy **(A)**. Intraoperative robot-assisted common bile duct exploration was facilitated by simultaneous visualization of the images from the choledochoscopy on the surgeon’s console **(B)**.

A 56-year-old woman with an 8-year history of hypothyroidism treated with methimazole underwent robotic left hepatectomy with intraoperative common bile duct exploration for multiple left intrahepatic duct stones associated with chronic cholangiohepatitis. A preoperative CT scan demonstrated multiple radioopaque stones in the left hepatic duct with ductal dilatation and parenchymal atrophy of the left lobe of the liver (Figure 4A).

Robotic left hepatectomy was performed as previously described [19]. In brief, the left lobe of the liver was freed from its attachments and adhesions due to chronic inflammation. The left hepatic artery was identified during hilar dissection and ligated. After identifying and preserving the venous branches to the caudate and Spiegel lobes, the left portal vein was ligated and divided. During parenchymal dissection, the left hepatic duct was identified and ligated. At this point, an incision was made proximal to the ligation of the left hepatic duct, and common bile duct exploration was performed.

The patient-side assistant introduced a 5-mm flexible choledochoscope (Olympus, Tokyo, Japan) into the abdominal cavity via the 12-mm assist port. The surgeon sitting at the master console directed the choledochoscope using the first and third arms of the robot. The Cadiere forceps controlled by the third robotic arm held open the left hepatic duct, while the bipolar Maryland dissector was used to direct the choledochoscope into the open stump of the left hepatic duct and into the common bile duct. The view obtained from the choledochoscope was made available to the surgeon sitting at the console via the Tilepro multi-input system simultaneously with an intraoperative 3D view obtained by the da Vinci System’s camera (Figure 4B). Robot-assisted intraoperative common bile duct exploration completely visualized the common bile duct and identification of the ampula of Vater but found no stones. Once the exploration was complete, the choledochoscope was removed, and the left hepatic duct was sutured closed using 5-0 prolene suture with robotic assistance (Additional file 3). Left hepatectomy was completed by identification and ligation of the left hepatic vein using a vascular endolinear stapler. The operation time was 465 minutes. The patient’s postoperative course was complicated by fluid collection at the hepatic resection bed, which did not require invasive intervention. The patient was discharged 12 days after surgery. Final pathological examination revealed hepatolithiasis with pigment stones and chronic proliferative cholangitis. A follow-up CT scan obtained 1 month after surgery confirmed that no radioopaque stones were left in the common bile duct.

## Discussion

The three complex robotic abdominal operations were successfully completed without difficulty or intraoperative complications. The use of the Tilepro to simultaneously visualize the images from the colonoscope, gastroscope, and choledochoscope made it possible to perform additional intraoperative endoscopic procedures without extra monitors or interference with the operations. The surgeons were able to continue with the operative procedures without distraction from their 3D view from the surgeon’s console.

We describe a novel application of integrating intraoperative endoscopy during robotic abdominal operations using a multi-input display program available on the da Vinci Surgical System. Three different intraoperative endoscopic procedures were successfully delivered to the surgeon’s console during robotic left hepatectomy, low anterior resection, and radical gastrectomy by employing the Tilepro, a multi-input display program. These three surgeries demonstrate the safety and feasibility of using the Tilepro to provide simultaneous, one-screen views of endoscopic procedures during robotic abdominal surgery, including rectal, gastric, and hepatobiliary operations.

Robotic operations have demonstrated safety and feasibility in various fields [20-22]. Robotic surgery offers the surgeon the potential to perform complex operations with increasing ease and precision as well as a quicker learning curve and adaptation to minimally invasive surgery over conventional laparoscopy [23,24]. Patients have benefited by decreased blood loss, decreased hospital stay, decreased pain, and increased satisfaction after their robotic procedures [5,8,9,25-27]. The advantages of robotic technology over conventional laparoscopic instruments have been predominantly attributed to the 3D operative view, tremor filter, 7 degrees of endowrist function, and control of four arms by the surgeon [5,28].

As experience increases with the use of robotic surgical platforms, additional advantages are being realized. Recently, a group of urologists used Tilepro for image-guided surgery during robotic nephrectomies [29]. Patient specific information such as preoperative CT scans and intraoperative ultrasound was viewed intraoperatively on the surgeon’s console to assist decision making during key portions of the operations. A multi-input display system permits the surgeon to view preoperative radiological images during surgery for guidance during the procedure.

Our study demonstrates another advantage of the surgical robotic platform: the ability of the surgeon to access various endoscopic images from the colonoscopy, gastroscopy, and choledochoscopy simultaneously during complex intraabdominal operations. This function shows the versatility of the multi-input display program and the ability of the surgeon to command a wider range of patient-specific information during the operation at his master console. No additional monitors are needed for the endoscopic views, not requiring the surgeon to stop the intraperitoneal portion of the operations. In fact, two views can be seen by the assistant and the scrub nurse on the assistant monitors, enabling everyone involved in the operation to share the same view. This function is especially useful during robot-assisted common bile duct exploration, where the surgeon was able to control the choledochoscope with the robotic arms without the need for another skilled assistant. The ability to perform both laparoscopy and choledochoscopy simultaneously using the current robotic surgical system demonstrates the potential for the development of a new integrated robotic surgery platform that allows the surgeon to simultaneously control two different procedures on one console. Since our study is an introduction of initial successful application of multi-image display system, we could not evaluate its clinical impact of by comparing with and without this new system. Apart from our study, this multi-image display system has innate limitations such as transmission failure due to a cabling problem [30].

## Conclusions

We present a novel use of the multi-input display program on the da Vinci Surgical System to facilitate the performance of intraoperative endoscopies during complex robotic operations. Our study offers another potentially beneficial application of the robotic surgery platform toward integration and simplification of combining additional procedures with complex minimally invasive operations.

## Competing interests

The authors declare that they have no competing interests.

## Authors’ contributions

YW contributed to the acquisition of data, analysis and interpretation of the data, drafted the manuscript, revised it with critical input, and gave final approval of the version to the published. GHC contributed to the design of the study, the acquisition of the data, involved in drafting and critical review of the manuscript for intellectual content, and gave final approval of the version to be published. BSM contributed to the design of the study, the acquisition of the data, involved in drafting and critical review of the manuscript for intellectual content, and gave final approval of the version to be published. WJH contributed to the conception, design, writing, critical and intellectual input of the manuscript, acquisition and analysis of the data and gave final approval of the version to be published.

## Pre-publication history

The pre-publication history for this paper can be accessed here:

http://www.biomedcentral.com/1471-2482/14/13/prepub

## Supplementary Material

Additional file 1 Video of Tilepro use for intraoperative sigmoidoscopy to identify the proximal margin of resection during robotic low anterior resection for colon cancer. Click here for file

Additional file 2 Video of Tilepro use for intraoperative gastroscopy to identify the proximal margin of resection during distal gastrectomy for gastric cancer. Click here for file

Additional file 3 Video of Tilepro use for intraoperative choledochoscopy to identify intrahepatic stones during left hepatectomy for chronic cholangiohepatitis. Click here for file
